# Semantically Synchronizing Multiple-Camera Systems with Human Pose Estimation

**DOI:** 10.3390/s21072464

**Published:** 2021-04-02

**Authors:** Zhe Zhang, Chunyu Wang, Wenhu Qin

**Affiliations:** 1 School of Instrument Science and Engineering, Southeast University, Nanjing 210096, China; zhangzhecnjs@gmail.com; 2 Microsoft Research Asia, Beijing 100089, China; chnuwa@microsoft.com

**Keywords:** multiple-camera system, temporal synchronization, epipolar geometry, human pose estimation

## Abstract

Multiple-camera systems can expand coverage and mitigate occlusion problems. However, temporal synchronization remains a problem for budget cameras and capture devices. We propose an out-of-the-box framework to temporally synchronize multiple cameras using semantic human pose estimation from the videos. Human pose predictions are obtained with an out-of-the-shelf pose estimator for each camera. Our method firstly calibrates each pair of cameras by minimizing an energy function related to epipolar distances. We also propose a simple yet effective multiple-person association algorithm across cameras and a score-regularized energy function for improved performance. Secondly, we integrate the synchronized camera pairs into a graph and derive the optimal temporal displacement configuration for the multiple-camera system. We evaluate our method on four public benchmark datasets and demonstrate robust sub-frame synchronization accuracy on all of them.

## 1. Introduction

Human-centred computer vision tasks and applications, e.g., pedestrian detection [1,2,3], 3D human pose estimation [4,5,6,7,8,9] and body reconstruction [10], benefit from multiple-camera systems. Multiple cameras provide different views from a set of angles of the same moment, which expands coverage and mitigates the occlusion problem in single-camera systems. In tightly controlled laboratory setups, it is possible to use industry-level cameras with high-end image capture devices and have them be temporally synchronized. However, such cameras and capture devices are typically costly and are not affordable for commercial-level applications at scale, for example, smart stores [11] with tens of, or even more, cameras.

To temporally synchronize multiple cameras, previous works often need additional external signals as references. For example, Joo et al. [12] uses hardware clocks to simultaneously trigger cameras with expensive equipment. Besides special hardware, moving a pattern board [13] or an LED in the scene are often used as the isolated synchronization stage. Blank frames caused by flash [14] can also be used as temporal reference cues. However, there is no semantic feature to differentiate between blank frames, which will fail for large temporal displacement. To free multiple-camera systems from external signals, Sinha et al. [15] and Takahashi et al. [16] explore the use of human body features from the videos to temporally calibrate multiple cameras for human-centered tasks. Additionally, compared to non-semantic signals such as blank frames, human-related features are semantically differentiable along the temporal dimension, which supports even long-range temporal displacement.

In this work, we address the problem of temporally calibrating multiple-camera systems for human-centred tasks using only human pose predictions from an off-the-shelf pose estimator. Our approach needs no training stage and can be directly applied to any multiple-camera system with no additional fine-tuning step. It achieves sub-frame accuracy for wide-range scenarios, including indoor and outdoor, single person and multiple persons, and even when heavy occlusion occurs. Besides this, our approach can be used both as a calibrating stage beforehand or as a sentry to monitor the system synchronization status during its run. In our approach, camera projective parameters are assumed to be known, which is often the case in real-world applications. In common applications, for example, motion capture and smart stores, cameras are typically fixedly installed on rigs or ceilings, and their projective parameters are also fixed. In practice, we observed that the temporal unalignment of multiple cameras is usually caused by inaccurate camera internal time information. To temporally synchronize them, we need to find the temporal displacements between each pair of cameras.

**Figure 1:**
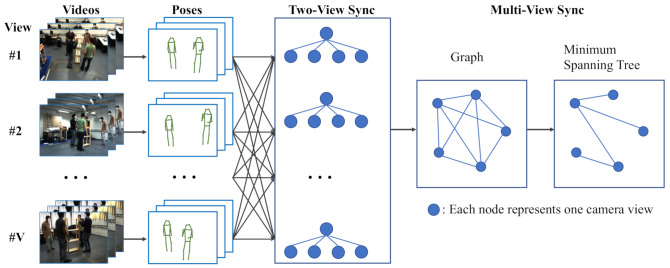
Overview of our method. V is number of camera views (V = 5 in this illustration). It first takes video frames from multiple views as input and outputs 2D human poses using an out-of-the-shelf HigherHRNet [17]. Then, we find the optimal offset and the corresponding minimal energy between each pair of views by the *two-view synchronization* method (Section 4). Finally, we form a fully connected graph whose nodes are camera views and derive the optimal offsets’ configuration for the multiple-camera system by minimum spanning tree algorithm (Section 5). Human faces are obfuscated to protect subjects’ privacy.

Figure 1 gives an overview of our approach. Firstly, the frames of all camera views are fed into an off-the-shelf pose estimator, such as HigherHRNet [17], to obtain pose predictions. The pose estimation network parameters are trained on COCO [18], which is a widely used human pose benchmark dataset. The parameters are *not* fine-tuned on any of our test datasets; therefore, the predicted poses may have many noises. Later in our method, we mitigate the effect of noise by leveraging a statistical algorithm on multiple frames. Then, we propose the measurement of pose distances from different views with epipolar distance (Section 3.2) and find the temporal displacement for each pair of views by minimizing an energy function related to epipolar distance. We propose a simple but effective multiple-person association algorithm to find the corresponding poses of a person in other views for epipolar distance computation. To make the most of the predicted poses, we devise a confidence score regularized energy function. The score is the maximum response value of the heatmap output by the pose estimator. We experimentally find that the improved energy function works robustly even for poor pose-prediction qualities. Finally, we construct a graph for the multiple-camera system. Each node of the graph represents one camera view. The edges linking nodes are the energy functions’ minimum values for each pair of camera views, as each node may connect multiple other nodes and lead to inconsistency in temporal displacement. The optimal displacement configuration for the whole multiple-camera system is then derived by the minimum spanning tree algorithm.

We evaluate our approach on four public multiview datasets including Human3.6M [19], Shelf [20], Campus [20] and Store [11]. These datasets provide temporally aligned multiview videos. We manually offset the videos and test our approach. Robust and sub-frame temporal synchronization accuracy are demonstrated on all of them.

Our contributions can be summarized as: (1) We provide a new framework to synchronize multiple cameras by exploiting semantic human features, i.e., human poses. It needs no training and is easy to use; (2) We propose a score-regularized method to make effective use of pose predictions to achieve stable and accurate results; (3) We demonstrate the effectiveness of our method on four representative benchmark datasets covering a wide-range of scenarios.

The rest of the paper is organized as follows. In Section 2, we discuss the related work. Section 3 introduces the basics for epipolar distance. Then, we describe how we synchronize a pair of cameras via minimizing the energy function related to epipolar distance in Section 4. In Section 5, we integrate all camera pairs into a graph and derive an optimal configuration for the multiple-camera system. In Section 6 and Section 7, we introduce the experimental datasets, evaluation metrics and results. Finally, we discuss the results in Section 8 and conclude this work in Section 9.

## 2. Related Work

### 2.1. Multiple-Camera System Synchronization

We briefly classify a temporal synchronization method for multiple-camera systems into two classes. The first class is based on external reference signals. The global hardware clock signal is the most widely used reference to trigger cameras at the same time [12]. Such systems require advanced cameras and capture devices, which are often very expensive. Other than hardware signals, additional visual signals can also be used. Moving a pattern board [13] or an LED in the scene are often used as an isolated synchronization stage before video capturing. Prarthana et al. [14] present a synchronization method based on blank frames caused by flash. The blank frames are detected and compared in the recordings to determine the temporal displacements between cameras. However, the flash could be annoying when recording, and it will fail if the temporal displacement is larger than the flash interval because there are no semantic features to differentiate between blank frames. Audio pattern [21,22] is another type of reference source. However, it first needs to align the video and audio, and then carefully compensate for the sound time-of-flight delay.

The second class analyzes internal video attributes, for example, human poses [16] or balls [23]. It eliminates the need for additional signals of isolated synchronization steps. Tamaki et al. [23] detect the table tennis ball in videos and utilize them to establish temporal correspondences between cameras. Takahashi et al. [16] detect human poses in videos for temporal synchronization. They optimize parameters for all cameras at the same time. However, they discuss situations when only single person is present. When multiple persons are present, occlusion often happens, which may cause person identification to fail and lead to larger synchronization errors. Although our work also uses 2D human poses as corresponding points between cameras, it differentiates from Takahashi et al. [16], as (1) our method is applicable for both single person and multiple persons; (2) we validate our method on several real-world datasets covering a wide range of scenarios and we analyse factors that affect synchronization accuracy by ablation study; (3) we synchronize the multiple-camera system in two stages, i.e., two-view and multi-view synchronization, which significantly reduces the computational complexity compared to optimizing all cameras at the same time.

### 2.2. 2D Human Pose Estimation

With the development of convolutional neural networks and a large dataset like COCO [18], MPII [24] and AIC [25], 2D human pose estimation obtains significant improvements. 2D human pose estimators are generally classified into two types: top-down and bottom-up methods. Top-down methods [26,27,28] firstly use a detector to localize the human in the image. Then, the image patches for each person are fed into a pose-estimation backbone network to obtain the intermediate pose representation, i.e., heatmaps. Finally, human poses are derived from the heatmaps by finding the maximum or coordinate integration [29]. Bottom-up methods [17,30,31] directly detect human joints on the whole image. Then, the same person’s joints are linked by PAF [30] or associative embeddings [17,31] to construct poses for each person. Besides the human poses, the maximum responses of the heatmaps are typically used as a threshold to determine if one pose is valid. The typical value for the threshold is 0.2. However, we observed that it conveys more information and can reflect the prediction quality to some extent. Thus, we explore its use as a score to regularize the energy function in this work.

## 3. The Basics for Multiview Geometry

We first introduce the basics of epipolar geometry to lay the groundwork. In particular, we discuss how the distance metric between two points can be computed from different viewpoints. Obtaining the distance between two points which are on the same view is straightforward. L2 norm, also known as Euclidean distance, are often used as the distance metric. However, if the two points are on different views, there is no direct way to calculate the Euclidean distance. With the help of camera parameters, we propose the use of *epipolar distance* as the distance metric for points across views. We first introduce epipolar geometry in Section 3.1 to illustrate the point-line correspondence between views. Then, in Section 3.2, we describe how to extend Euclidean distance for points across views.

### 3.1. Epipolar Geometry

**Figure 2:**
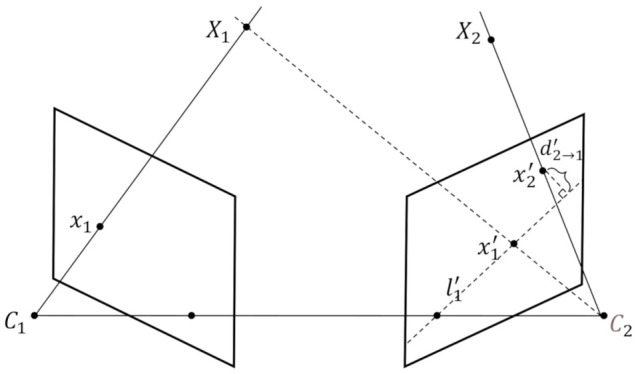
Illustration for epipolar geometry.

Let us denote a point in 3D space as $X\in R^{4\times 1}$, as shown in Figure 2. This could be the location of a 3D body joint in the context of pose estimation. Note that homogeneous coordinate and column vectors are used to represent a point. The 3D point $X_{1}$ is imaged in two camera views, at $x_{1}=PX_{1}$ in the first, and ${x_{1}}^{\prime }=P^{\prime }X_{1}$ in the second, where $x_{1}$ and ${x_{1}}^{\prime }\in R^{3\times 1}$ represent 2D points in images, P and $P^{\prime }\in R^{3\times 4}$ are the projection matrix for each camera. In practice, we have the 2D measurement $x_{1}$ for the 3D point $X_{1}$, but the depth is unknown. If we assume that $X_{1}$ could be at any depth, the corresponding point ${x_{1}}^{\prime }$ for $x_{1}$ could move freely on the line ${l_{1}}^{\prime }$ on the second view. The line ${l_{1}}^{\prime }$ on the second view is the corresponding *epipolar line* for $x_{1}$ of the first view. On the contrary, given ${x_{1}}^{\prime }$ of the second view, we can also find an epipolar line $l_{1}$ on the first view. In particular, the epipolar line $l_{1}$ and ${l_{1}}^{\prime }$ are computed as

(1) $$\begin{array}{cc} \; & {l_{1}}^{\prime }=Fx_{1}\\ \; & l_{1}=F^{⊤}{x_{1}}^{\prime }, \end{array}$$

where $F\in R^{3\times 3}$ is the fundamental matrix, which can be derived from P and $P^{\prime }$. Readers can refer to [32] for a detailed derivation.

### 3.2. Distance Metric

Given 2D measurements $x_{1}$ and ${x_{2}}^{\prime }$ on the first and second view, respectively, although we cannot compute the direct Euclidean distance between this two points, an indirect distance metric could be obtained by utilizing the point-line correspondence described in the previous section. As shown in Figure 2, the distance from ${x_{2}}^{\prime }$ to $x_{1}$ can be represented by the point-to-line distance $d_{2\to 1}^{\prime }$ on the second view, because the mapping between ${l_{1}}^{\prime }$ and $x_{1}$ is one-to-one. Conversely, the indirect distance from $x_{1}$ to ${x_{2}}^{\prime }$ can be represented by $d_{1\to 2}$ on the first view. Mathematically, $d_{2\to 1}^{\prime }$ and $d_{1\to 2}$ are

(2) $$\begin{array}{cc} d_{2\to 1}^{\prime } & =\frac{|{x_{2}}^{\prime }{}^{⊤}Fx_{1}|}{\sqrt{{\left(Fx_{1}\right)}_{1}^{2}+{\left(Fx_{1}\right)}_{2}^{2}}}\\ d_{1\to 2} & =\frac{|{x_{1}}^{⊤}F^{⊤}{x_{2}}^{\prime }|}{\sqrt{{\left(F^{⊤}{x_{2}}^{\prime }\right)}_{1}^{2}+{\left(F^{⊤}{x_{2}}^{\prime }\right)}_{2}^{2}}} \end{array}$$

where F is the fundamental matrix, the subscript 1 or 2 denotes the first or second element of a vector. We know ${\left({x_{2}}^{\prime }{}^{⊤}Fx_{1}\right)}^{⊤}={x_{1}}^{⊤}F^{⊤}{x_{2}}^{\prime }$; therefore, it is clear $d_{2\to 1}^{\prime }$ is not equal to $d_{1\to 2}$. To make the distance metric symmetrical, we propose merging the denominators of the two formulas as

(3) $$d_{1↔2}=\frac{|{x_{2}}^{\prime }{}^{⊤}Fx_{1}|}{\sqrt{{\left(Fx_{1}\right)}_{1}^{2}+{\left(Fx_{1}\right)}_{2}^{2}+{\left(F^{⊤}{x_{2}}^{\prime }\right)}_{1}^{2}+{\left(F^{⊤}{x_{2}}^{\prime }\right)}_{2}^{2}}}$$

We call the distance in Equation (3) the *epipolar distance* in this paper. The epipolar distance is symmetrical. In addition, we can directly calculate the distance between a pair of 2D locations without knowing their corresponding 3D points.

In the context of this paper, the 2D points $x_{1}$ and ${x_{2}}^{\prime }$ are detections of human body keypoints of same type, but may be of different time instants from two views. Ideally, if the measurement is noise-free, and the videos are temporally aligned, the epipolar distance should be 0. Otherwise, if the human moves in a constant direction, the epipolar distance grows with temporal unalignment.

## 4. On Two-View Synchronization

We first describe how to solve the two-view synchronization problem by minimizing an energy function related to Epipolar distance. Then, we incorporate 2D pose confidence into this framework to obtain improved accuracy and robustness. Finally, we describe the implementation details.

### 4.1. Problem Formulation

We represent one 2D human pose by a set of *K* joints, as $P=\{J^{\left(1\right)},J^{\left(2\right)},\cdots ,J^{\left(K\right)}\}$; *K* is the number of total joints. Each *J* represents the state of a joint, such as its 2D location on the image, as $J^{\left(k\right)}=\left(x^{\left(k\right)},y^{\left(k\right)}\right),k\in \left[1,K\right]$. Besides this, we denote view index, frame index and person index in right-subscript. For example, $P_{\left(v,t,i\right)}$ represent the *i*-th human pose of the *v*-th camera view’s *t*-th frame.

Ideally, if the videos captured by the two camera views are temporally synchronized and the 2D pose estimation results are accurate, the Epipolar distance between one person’s poses in the two views of the same frame index should be zero. Therefore, we propose an energy function E related to the epipolar distances between the two videos, and minimize the energy function to find the best temporal offset for two-view synchronization. The energy function is

(4) $$E\left(\Delta t\right)=\frac{1}{Z}\sum_{t=1}^{T}\sum_{i=1}^{N_{t}}\sum_{k=1}^{K}\delta d\left(J_{\left(v_{1},t,i\right)}^{\left(k\right)},J_{\left(v_{2},t+\Delta t,i\right)}^{\left(k\right)}\right),$$

where Z is partition function related to total number of joint pairs; *T* represents number of overlapped frames after offsetting the second video Δt frames; $N_{t}$ represents the number of humans in the *t*-th frame; d(·,·) is the epipolar distance of a pair of joints from two views; δ is the filter function which outputs 0 or 1 to indicate if a pair of joints is valid. Following common practice in 2D human pose estimation works, we regard those joints whose confidence score of less than 0.2 as invalid. We then find the best temporal offset $\Delta t^{*}$ by

(5) $$\Delta t^{*}=\underset{\Delta t}{\arg\min}\;E\left(\Delta t\right).$$

### 4.2. Multiple Person Association across Cameras

One limitation is that we could hardly know the personal correspondence among different views. Human pose estimators are usually trained on the MPII [24], COCO [18] or AIC [25] dataset, neither of which is provided with personal identity information for multiview association. To our knowledge, none of the out-of-the-shelf 2D human pose estimation networks atr equipped with a person identification module. This imposes challenges when computing energy function in Equation (4). Fortunately, due to the sparsity and diversity of human poses, the epipolar distances between non-corresponding poses are statistically much larger than the corresponding pose pairs. To bypass the complex person identification problem, we simply regard the one whose pose has the least average epipolar distance between other views as the corresponding pose, as in

(6) $$\mathrm{Corresponding}\;\mathrm{ID}=\underset{i_{2}}{\arg\min}\sum_{k=1}^{K}\delta d\left(J_{\left(v_{1},t,i\right)}^{\left(k\right)},J_{\left(v_{2},t+\Delta t,i\right)}^{\left(k\right)}\right).$$

**Figure 3:**
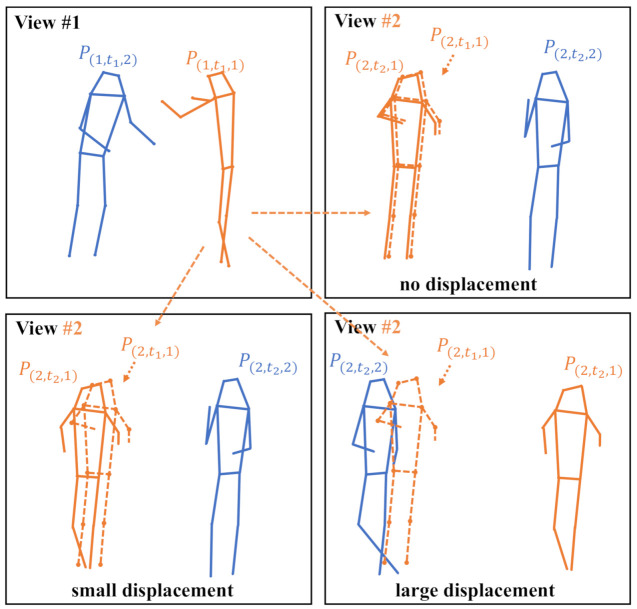
Illustration of multiple person association typical cases. The two frames are from two different camera views of the *same* or *different* time instant. To find the corresponding pose of the same person in the view #2 for pose $P_{\left(1,t_{1},1\right)}$ of view #1, we compute the distances between all poses in view #2 and $P_{\left(1,t_{1},1\right)}$. Then, the pose of least distance to $P_{\left(1,t_{1},1\right)}$ is selected as the correspondence. Note that $P_{\left(2,t_{1},1\right)}$ of the dashed line is a proxy for $P_{\left(1,t_{1},1\right)}$, which is only for illustration. When the two frames are of the same time instant (upper-right), it finds the correct corresponding pose and the distance is very small. When there are small temporal displacements (lower-left), it is likely that the correct correspondence will be found, but the distance will be a little larger. When the temporal displacement is large (lower-right), the wrong correspondence could be assigned and the distance is generally much larger.

We illustrate this approach in Figure 3. Although this approach is very simple, the experimental results validate that it works pretty well.

### 4.3. Score Regularization

Together with the 2D pose coordinates, the human pose estimator also outputs a confidence score for each joint to reflect its detection quality. Previous works often calculate the average score of all joints for a human proposal, to determine if it is a false positive threshold. We find that it, in fact, conveys more information, which has not been effectively utilized. Due to the different levels of occlusion, background clutter and light variance, 2D human pose results have different noise levels. We observe that the confidence score can indicate the noise to a certain extent. However, in Equation (4), all poses are regarded as being of the same precision. In this section, we propose regularizing the energy function with the confidence score as

(7) $$E\left(\Delta t\right)=\frac{1}{Z}\sum_{t=1}^{T}\sum_{i=1}^{N_{t}}\sum_{k=1}^{K}\min\left(\Lambda_{\left(v_{1},t,i\right)}^{\left(k\right)},\Lambda_{\left(v_{2},t+\Delta t,i\right)}^{\left(k\right)}\right)\delta d\left(J_{\left(v_{1},t,i\right)}^{\left(k\right)},J_{\left(v_{2},t+\Delta t,i\right)}^{\left(k\right)}\right),$$

where $\Lambda_{\left(v_{1},t,i\right)}^{\left(k\right)}$ and $\Lambda_{\left(v_{2},t+\Delta t,i\right)}^{\left(k\right)}$ are the related confidence scores of joints $J_{\left(v_{1},t,i\right)}^{\left(k\right)}$ and $J_{\left(v_{2},t+\Delta t,i\right)}^{\left(k\right)}$.

### 4.4. Implementation Details

We use HigherHRNet [17] as the off-the-shelf, bottom-up 2D pose estimation backbone network. The parameters of the backbone network are provided by the HigherHRNet authors, who are trained on the COCO [18] dataset and *not* fine-tuned on any of our test datasets. The short side of input image is resized to 512 pixels and the aspect ratio stays the same.

## 5. On Multi-View Synchronization

In this section, we present the details of our method to derive the optimal temporal displacement configuration to construct separate cameras into a synchronized multiple-camera system.

### 5.1. Graph Based Global Minimization

Recall that we have already obtained the temporal displacement for every pair of camera views. The displacement for one view could be determined from multiple reference views. However, the displacements between reference views might conflict. Take three views as an example: the displacements for view pairs (1,2), (1,3) and (2,3) are 0, 2 and 4 frames. It would be hard to determine a consistent displacement configuration. In this case, we propose the use of a graph-based minimum spanning tree algorithm to find the global optimal displacement configuration of the least sum of epipolar distances.

We construct an undirected graph G=(V,E) on the multiple-camera system. See the right half of Figure 1. Each node in the vertex set $V=\{v_{i}\}$ represents a camera view. Each edge *e* in the edge set represents attributes between two camera views, e.g., temporal displacement, epipolar distance energy value E and total number of valid joints in the two videos. Finally, the minimum weight spanning tree is used to obtain the optimal temporal displacement configuration.

### 5.2. Implementation Details

Since the overlap space of some camera pairs is small, the number of valid joint pairs might be small and the corresponding temporal displacement for the camera pairs will also not be very accurate. We filter out the camera pairs of a small number of valid joint pairs by a threshold predefined or adaptively assigned as Q1 quantile of all pairs. Energy function value E is used as the edge weight in a minimum weight spanning tree. Kruskal algorithm [33] is used to obtain the minimum weight spanning tree.

## 6. Dataset and Metric

**Figure 4:**
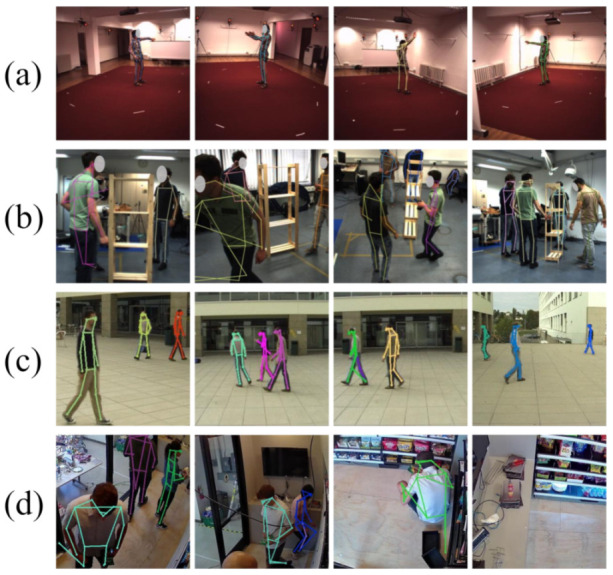
Sample images and human pose predictions from (**a**) Human3.6M, (**b**) Shelf, (**c**) Campus and (**d**) Store datasets. The poses could be noisy for challenging cases. Better viewed in color and zoomed-in. Human faces are obfuscated to protect subjects’ privacy.

We evaluate our method on four representative public datasets (see Figure 4) which covers diverse scenarios and situations. All these datasets provide intrinsic and extrinsic camera parameters.

### 6.1. The Human3.6M Dataset

This dataset [19] provides videos captured by four cameras. The videos are triggered by an external clock signal to ensure perfect synchronization. The frame rate is 50 fps. In each video, there is a single subject performing one of sixteen daily actions. There are seven subjects (1, 5, 6, 7, 8, 9, 11) in total. Each video contains about 1000 to 3000 frames.

### 6.2. The Campus Dataset

This dataset [20] captures three people walking and interacting with each other in an open outdoor square by three cameras. Each video contains 2000 frames. The scale of people in the image is much smaller than other datasets.

### 6.3. The Shelf Dataset

This dataset [20] captures four people interacting with each other and disassembling a shelf in a small indoor environment by five cameras. Each video contains 3200 frames. People are often occluded by other people or the shelf in the scene.

### 6.4. The Store Dataset

This dataset [11] evenly arranges twelve cameras on the ceiling of a store with shelves. Each camera captures a small part of the store from a height. The frame rate is 10 fps. Each video contains about 790 frames. There are, in total, five people in the scene.

### 6.5. The Temporal Displacement Metric

We introduce the synchronization error (SynErr) to measure temporal displacement between predicted offset and true offset over all video pairs for multiple camera systems. The SynErr in number of frames is as

(8) $$\mathrm{SynErr}=\frac{2}{V(V-1)}\sum_{i=1}^{V}\sum_{j=i+1}^{V}\left|R_{i\to j}-T_{i\to j}\right|,$$

where *V* is number of views in the multiple-camera system, $R_{i\to j}$ and $T_{i\to j}$ are, respectively, the predicted and true offset for a video pair of view *i* and view *j*. For synchronized video pairs, the true offset *T* is 0. The smaller the value of SynErr, the better the performance.

## 7. Experimental Results

Table 1 shows SynErr results on the four public datasets. We denote the baseline method proposed in Section 4.1 and the improved score regularized version in Section 4.3 as *Simple* and *Score*, respectively.

On Human3.6M and Campus datasets, the Simple method achieves sub-frame temporal displacement accuracy, while on Shelf and Store datasets, the errors become much larger. We attribute this to the 2D human pose qualities estimated by the out-of-the-shelf pose estimator and the length of videos. In Human3.6M, there is only a single subject in a laboratory capture room and the video lengths are generally longer. Therefore, Human3.6M obtains the least SynErr among all datasets. In Shelf and Store datasets, there is more occlusion, caused by both the subjects themselves and the objects in the scene, such as shelves. Consequently, the estimated human poses in these dataset suffer missing detection and lower precision, leading to larger errors.

With the help of human pose confidence scores as regularization terms in the energy function of Equation (7), the SynErr consistently decrease for the Score column of Table 1. The improvements are significant for Shelf and Store datasets. They reduce from 8.8 and 3.1 to 0 and 0.6, respectively. This indicates that the improved Score method works robustly, even when the human pose qualities are poor, by effectively utilizing the pose scores. The improvements are small for Human3.6M and Campus datasets, because they already work well with the Simple method.

### 7.1. Ablation Study on Number of Frames

**Figure 5:**
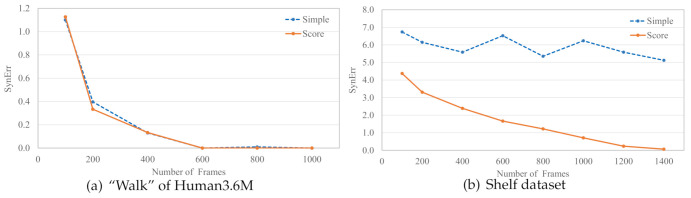
Results for Human3.6M “Walk” action (**a**) and Shelf (**b**) dataset when different numbers of frames are used. For each SynErr datapoint in the graph, we randomly crop the number of corresponding *x*-axis value frames from original videos and repeat the experiment 50 times to obtain the average SynErr.

Figure 5 shows ablation results on Human3.6M and Shelf datasets when a different number of frames is used for temporal displacement calibration. We choose the “Walk” action from Human3.6M, since it is the most common action in real-world applications. On Human3.6M (left figure), the SynErr difference between Simple and Score methods is negligible. This happens because the estimated pose accuracy is high, leaving little room for improvement. When 600 or more frames are used, the SynErr reaches its lower bound.

On Shelf (right figure), the estimated human poses contain more noises and errors. The SynErr hardly decreases, even when more frames are used for the Simple method. The Score method consistently reduce SynErr by effectively utilizing more frames. At about 1400 frames, the Score method reaches its lower bound error.

### 7.2. Ablation Study on Action Types

**Figure 6:**
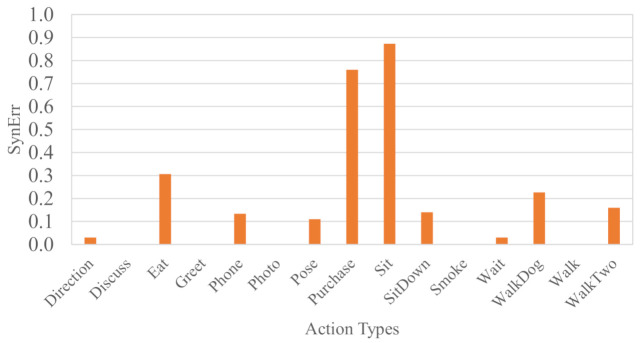
Results for different action types of Human3.6M dataset. The Score method is used. We randomly crop 600 frames from original videos and repeat the experiment for 50 times to obtain the average SynErr.

Figure 6 shows SynErr results of Score method for each action type of Human3.6M dataset. We randomly crop 600 frames from original videos and repeat the experiment for 50 times to obtain the average SynErr in the graph. “Purchase” and “Sit” actions have the largest SynErr among all actions. In these action videos, the subject does not often walk around in the room but stays still for most of the time. Such cases need more frames to reduce the SynErr. On the other hand, actions like “Walk”, “Smoke”, etc. achieves very low SynErr. In these action videos, the subjects often walk around and are in regular poses which are easy for the pose estimator. We conjecture that actions similar to “Walk” where subjects have stable global movements best fit our method.

## 8. Discussion

### 8.1. How Scores Work Effectively?

The results for *Score* method are superior to *Simple* method. It is not only more accurate but also more robust for various scenarios. This should be attributed to the effective usage of scores in Equation (7). The scores are the corresponding maximum response values of heatmaps. They are typically used for threshold to determine if a joint detection is valid. However, we observed that the scores actually convey more information about detection quality, and were not effectively used in previous works. We borrow the data fusion idea from Kalman filter [34]. We regard human poses as 2D location measurements and we assume the measurement noise is Gaussian noise. Then, the confidence score can be seen as an approximation of the reciprocal of noise variance. Ideally, one pair of joints could determine the energy function. However, the signal to noise ratio (SNR) will be very low because of imperfect human pose estimation. By merging all the joint pairs into the energy function with appropriate weights, similar to the Kalman filter, the noise level of final energy function is lower than *any* of the separate joint pairs. Therefore, our *Score* method is accurate and robust.

### 8.2. What Kind of Actions Are Suitable?

As shown in the experimental results of Human3.6M in Figure 6 and benchmark datasets in Table 1, our method works well for all kind of action. Nevertheless, we find that, for some actions, fewer video frames are used to reach a similar accuracy to other actions. Actions similar to “Walk” are more suitable for our method. We attribute this to the characteristic energy function in Equations (4) and (7). When the subjects move stably, the energy also grows smoothly as a temporal offset. Such actions contain abundant information for synchronization. On the contrary, in actions like “Sit”, the movement is mainly local motion. They contain little information and more frames are needed to obtain feasible performance. In practice, subjects may perform a series of action types in series. For example, a shopper walks to a shelf (global motion) and picks up a commodity (local motion). To ensure our method makes use of all kind of actions, we could detect subjects’ actions and classify them, to assign adaptive weights to the clips for better synchronization. Since this is beyond this paper, we leave this for our future work.

## 9. Conclusions

This paper presents a novel framework to semantically synchronize multiple-camera systems with human pose estimation. It features out-of-the-box usage and robust and accurate sub-frame performance. It firstly synchronizes every pair of cameras in the system by minimizing an energy function. Then, the optimal temporal displacement configuration is derived by the graph algorithm. We evaluate the approach on four widely used benchmark datasets and show consistent sub-frame accuracy. We discuss how relevant factors such as number of frames and action type possibly affect the performance by ablation study. We also explain our insight into using the scores for regularization. As the subjects naturally perform a series of different actions and some actions are more informative for synchronization, our future work will focus on how to detect those segments from long videos.

## Figures and Tables

**Table 1:** SynErr results on Human3.6M, Campus, Shelf and Store datasets.

Dataset	*Simple*	*Score*
Human3.6M	0.1	0.0
Campus	0.7	0.7
Shelf	8.8	0.0
Store	3.1	0.6

## Data Availability

Not applicable.

## References

[1] Hou Y., Zheng L., Gould S. Multiview detection with feature perspective transformation. Proceedings of the 16th European Conference.

[2] Mittal A., Davis L.S. (2003). M_2_Tracker: A multi-view approach to segmenting and tracking people in a cluttered scene. Int. J. Comput. Vis..

[3] Fang Z., Vázquez D., López A.M. (2017). On-board detection of pedestrian intentions. Sensors.

[4] Zhang Z., Wang C., Qiu W., Qin W., Zeng W. (2020). AdaFuse: Adaptive Multiview Fusion for Accurate Human Pose Estimation in the Wild. Int. J. Comput. Vis..

[5] Zhang Z., Wang C., Qin W., Zeng W. Fusing Wearable IMUs with Multi-View Images for Human Pose Estimation: A Geometric Approach. Proceedings of the 2020 IEEE/CVF Conference on Computer Vision and Pattern Recognition (CVPR).

[6] Qiu H., Wang C., Wang J., Wang N., Zeng W. (2019). Cross View Fusion for 3D Human Pose Estimation. Proceedings of the IEEE/CVF International Conference on Computer Vision (ICCV).

[7] Tu H., Wang C., Zeng W. VoxelPose: Towards Multi-Camera 3D Human Pose Estimation in Wild Environment. Proceedings of the 16th European Conference.

[8] Xie R., Wang C., Wang Y. Metafuse: A pre-trained fusion model for human pose estimation. Proceedings of the IEEE/CVF Conference on Computer Vision and Pattern Recognition (CVPR).

[9] Liu P., Zhang Z., Meng Z., Gao N. (2021). Monocular Depth Estimation with Joint Attention Feature Distillation and Wavelet-Based Loss Function. Sensors.

[10] Saito S., Huang Z., Natsume R., Morishima S., Kanazawa A., Li H. Pifu: Pixel-aligned implicit function for high-resolution clothed human digitization. Proceedings of the IEEE/CVF International Conference on Computer Vision (ICCV).

[11] Chen L., Ai H., Chen R., Zhuang Z., Liu S. Cross-View Tracking for Multi-Human 3D Pose Estimation at over 100 FPS. Proceedings of the IEEE/CVF Conference on Computer Vision and Pattern Recognition (CVPR).

[12] Joo H., Simon T., Li X., Liu H., Tan L., Gui L., Banerjee S., Godisart T., Nabbe B., Matthews I. (2019). Panoptic studio: A massively multiview system for social interaction capture. IEEE Trans. Pattern Anal. Mach. Intell..

[13] Zhang Z. Flexible camera calibration by viewing a plane from unknown orientations. Proceedings of the Seventh IEEE International Conference on Computer Vision.

[14] Shrestha P., Weda H., Barbieri M., Sekulovski D. Synchronization of multiple video recordings based on still camera flashes. Proceedings of the 14th ACM International Conference on Multimedia.

[15] Sinha S.N., Pollefeys M., McMillan L. Camera network calibration from dynamic silhouettes. Proceedings of the 2004 IEEE Computer Society Conference on Computer Vision and Pattern Recognition.

[16] Takahashi K., Mikami D., Isogawa M., Kimata H. Human pose as calibration pattern; 3D human pose estimation with multiple unsynchronized and uncalibrated cameras. Proceedings of the 2018 IEEE/CVF Conference on Computer Vision and Pattern Recognition Workshops (CVPRW).

[17] Cheng B., Xiao B., Wang J., Shi H., Huang T.S., Zhang L. HigherHRNet: Scale-Aware Representation Learning for Bottom-Up Human Pose Estimation. Proceedings of the 2020 IEEE/CVF Conference on Computer Vision and Pattern Recognition (CVPR).

[18] Lin T.Y., Maire M., Belongie S., Hays J., Perona P., Ramanan D., Dollár P., Zitnick C.L. Microsoft coco: Common objects in context. Proceedings of the 13th European Conference.

[19] Ionescu C., Papava D., Olaru V., Sminchisescu C. (2014). Human3.6m: Large scale datasets and predictive methods for 3D human sensing in natural environments. IEEE Trans. Pattern Anal. Mach. Intell..

[20] Belagiannis V., Amin S., Andriluka M., Schiele B., Navab N., Ilic S. 3D pictorial structures for multiple human pose estimation. Proceedings of the 2014 IEEE Conference on Computer Vision and Pattern Recognition.

[21] Shrstha P., Barbieri M., Weda H. Synchronization of multi-camera video recordings based on audio. Proceedings of the 15th ACM International Conference on Multimedia.

[22] Hasler N., Rosenhahn B., Thormahlen T., Wand M., Gall J., Seidel H.P. Markerless motion capture with unsynchronized moving cameras. Proceedings of the 2009 IEEE Conference on Computer Vision and Pattern Recognition.

[23] Tamaki S., Saito H. (2015). Reconstructing the 3D Trajectory of a Ball with Unsynchronized Cameras. Int. J. Comput. Sci. Sport.

[24] Andriluka M., Pishchulin L., Gehler P., Schiele B. 2D Human Pose Estimation: New Benchmark and State of the Art Analysis. Proceedings of the 2014 IEEE Conference on Computer Vision and Pattern Recognition.

[25] Wu J., Zheng H., Zhao B., Li Y., Yan B., Liang R., Wang W., Zhou S., Lin G., Fu Y. (2017). Ai challenger: A large-scale dataset for going deeper in image understanding. arXiv.

[26] Xiao B., Wu H., Wei Y. Simple baselines for human pose estimation and tracking. Proceedings of the 15th European Conference.

[27] Sun K., Xiao B., Liu D., Wang J. Deep High-Resolution Representation Learning for Human Pose Estimation. Proceedings of the IEEE/CVF Conference on Computer Vision and Pattern Recognition (CVPR).

[28] Newell A., Yang K., Deng J. Stacked hourglass networks for human pose estimation. Proceedings of the14th European Conference.

[29] Sun X., Xiao B., Wei F., Liang S., Wei Y. Integral human pose regression. Proceedings of the European Conference on Computer Vision (ECCV).

[30] Cao Z., Simon T., Wei S.E., Sheikh Y. Realtime multi-person 2d pose estimation using part affinity fields. Proceedings of the IEEE Conference on Computer Vision and Pattern Recognition (CVPR).

[31] Newell A., Huang Z., Deng J. Associative embedding: End-to-end learning for joint detection and grouping. Proceedings of the 31st International Conference on Neural Information Processing Systems.

[32] Hartley R., Zisserman A. (2003). Multiple View Geometry in Computer Vision.

[33] Kruskal J.B. (1956). On the shortest spanning subtree of a graph and the traveling salesman problem. Proc. Am. Math. Soc..

[34] Kalman R.E. (1960). A New Approach to Linear Filtering And Prediction Problems. ASME J. Basic Eng..

